# Identification of prognostic and therapeutic value of CC chemokines in Urothelial bladder cancer: evidence from comprehensive bioinformatic analysis

**DOI:** 10.1186/s12894-021-00938-w

**Published:** 2021-12-10

**Authors:** Yuxin Li, Xiong Chen, Dongjie Li, Zhiming Yang, Yao Bai, Sheng Hu, Zhenyu Liu, Jie Gu, XiaoBo Zhang

**Affiliations:** 1 Department of Geriatric Urology, Xiangya International Medical Center, Xiangya Hospital, Central South University, Changsha, Hunan 410008 People’s Republic of China; 2 National Clinical Research Center for Geriatric Disorders, Xiangya Hospital, Central South University, Changsha, Hunan 410008 People’s Republic of China; 3 Urolithiasis Institute of Central South University, Changsha, Hunan 410008 People’s Republic of China; 4 Martini-Klinik Prostate Cancer Center, University Hospital Hamburg-Eppendorf, Hamburg, Germany

**Keywords:** Bioinformatics analysis, CC chemokines, Urothelial bladder cancer, Biomarker, Prognosis

## Abstract

**Background:**

Urothelial bladder cancer (BC) is one of the most prevalent malignancies with high mortality and high recurrence rate. Angiogenesis, tumor growth and metastasis of multiple cancers are partly modulated by CC chemokines. However, we know little about the function of distinct CC chemokines in BC.

**Methods:**

ONCOMINE, Gene Expression Profiling Interactive Analysis (GEPIA), Kaplan–Meier plotter, cBioPortal, GeneMANIA, and TIMER were used for analyzing differential expression, prognostic value, protein–protein interaction, genetic alteration and immune cell infiltration of CC chemokines in BC patients based on bioinformatics.

**Results:**

The results showed that transcriptional levels of CCL2/3/4/5/14/19/21/23 in BC patients were significantly reduced. A significant relation was observed between the expression of CCL2/11/14/18/19/21/23/24/26 and the pathological stage of BC patients. BC patients with high expression levels of CCL1, CCL2, CCL3, CCL4, CCL5, CCL8, CCL13, CCL15, CCL17, CCL18, CCL19, CCL22, CCL25, CCL27 were associated with a significantly better prognosis. Moreover, we found that differentially expressed CC chemokines are primarily correlated with cytokine activity, chemokines receptor binding, chemotaxis, immune cell migration. Further, there were significant correlations among the expression of CC chemokines and the infiltration of several types of immune cells (B cells, CD8+ T cells, CD4+ T cells, macrophages, neutrophils, and dendritic cells).

**Conclusions:**

This study is an analysis to the potential role of CC chemokines in the therapeutic targets and prognostic biomarkers of BC, which gives a novel insight into the relationship between CC chemokines and BC.

## Background

Bladder cancer is one of the most prevalent cancers, with an estimated 549,000 new cases and 200,000 deaths reported in 2018 [1]. Urothelial bladder cancer accounts for more than 90% of bladder cancer [2]. Typically, the main therapy strategies of bladder cancer comprise Transurethral resection of bladder tumor (TURBT), radiotherapy, chemotherapy, and immunotherapy [3–6] and with limited survival [7]. Besides, the high recurrence rate (up to 60–70%) [6] of Bladder cancer patients pose a heavy load on public health system [8]. Moreover, cystectomy, as the most important method of tumor treatment, is an invasive procedure. Cystectomy was not promising to increase the recurrence-free survival (RFS) and overall survival (OS) in the expected range even with extended removal of lymph nodes [9].

Chemokines, constituting the largest family of cytokines, are chemotactic cytokines that mediate immune cell migration and lymphoid tissue growth [10]. Sequencing and gene expression studies have found the CC chemokines may play an important role in the tumorigenesis and progression of distinct tumors [11–13]. Previous studies have identified several CC chemokines were associated with disease-specific survival [14], tumor growth and progression [12]. Studies interpreted that CC chemokines may affect the abundance, infiltration and accumulation of immune cells [15, 16]. Thus, CC chemokines have multiple functions in tumor progression and invasion, and they serve as prognostic biomarkers for many types of tumors, including BC. However, the expression and prognostic values of CC chemokines in BC still remain unclear.

In this study, we performed a comprehensive analysis of CC chemokines to evaluate their potential value as therapeutic targets and prognostic biomarkers based on several large public databases, thus supplying informative assistance to help clinicians select appropriate therapeutic drugs and more accurately prognosis in BC patients.

## Methods

### ONCOMINE

The mRNA levels of distinct CC chemokines in diverse cancer types were analysed in ONCOMINE (www.oncomine.org), an online database providing powerful, genome-wide expression analysis with cancer microarray information [17]. In this study, a *p*-value < 0.05, a fold change of 2, and a gene rank in the top 10% were set as the significance thresholds. The mRNA expression of CC chemokines in clinical cancer specimens were compared with those in normal controls. Student’s t-test was used to analyze the difference in the expression of CC chemokines in BC.

### GEPIA

GEPIA (http://gepia.cancer-pku.cn/index.html) is a new analytical tool using a standard processing pipeline and consist of thousands of tumors and normal tissue samples data [18]. In this research, a differential gene expression analysis of mRNA expression of tumor and normal tissues, pathological stage analysis, and correlative prognostic analysis through GEPIA. Student’s t-test was used to generate a p-value for the expression or pathological stage analysis.

#### Kaplan–Meier plotter

The prognostic analysis of CC chemokines patients was also performed by using Kaplan–Meier plotter (http://kmplot.com/analysis/) [19], which is an online tool about the association of gene expression with the survival of patients. Data as the number-at-risk cases, median values of mRNA expression levels, HRs, 95% CIs and p-values can be obtained from the K–M plotter webpage. A statistically significant difference was considered when the *p*-value was < 0.05. Patient samples were split into two groups by median expression (high versus low expression) and assessed by a Kaplan–Meier survival plot.

#### cBioPortal

cBioPortal (www.cbioportal.org) is a comprehensive web resource, can visualize and analyze multidimensional cancer genomics data [20]. Based on The Cancer Genome Atlas (TCGA) database, genetic alterations, and co-expression of CC chemokines were obtained from cBioPortal.

#### String

STRING (https://string-db.org/) is a website that provides a comprehensive and objective global network of protein–protein interaction (PPI) [21]. A PPI network analysis was performed to collect and integrate the different expressions of CC chemokines and potential interactions through STRING.

#### GeneMANIA

GeneMANIA (http://www.genemania.org) is a website about gene information, analyzing gene lists and prioritizing genes for functional assays [22]. The potential interactions between different CC chemokines were analysed on it.

#### Timer

TIMER (https://cistrome.shinyapps.io/timer/) is web interface that provides systematic evaluations of the infiltration of different immune cells and their clinical impact [23]. In this study, “Gene module” was selected to evaluate the correlation between CC chemokines level and the infiltration of immune cells. “Survival module” was used to evaluate the correlation among clinical outcome and the infiltration of immune cells and CC chemokine expression.

## Results

### Expression of different CC chemokines BC

**Fig. 1:**
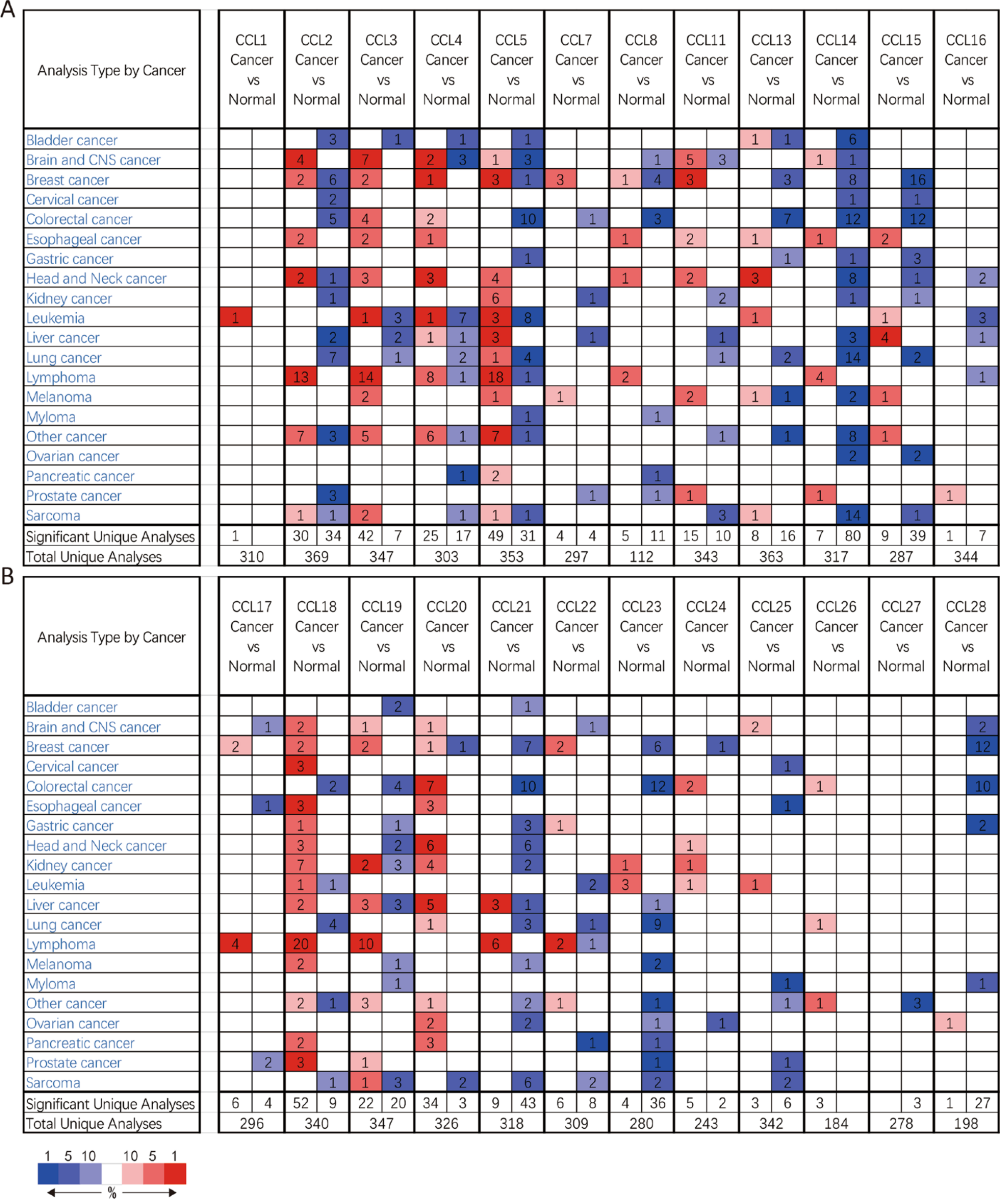
**A** CCL1-CCL16, **B** CCL17-CCL28 mRNA levels of CC chemokines in BC (ONCOMINE). The figure shows the numbers of datasets with statistically significant mRNA over-expression (red) or downregulated expression (blue) of CC chemokines

**Fig. 2:**
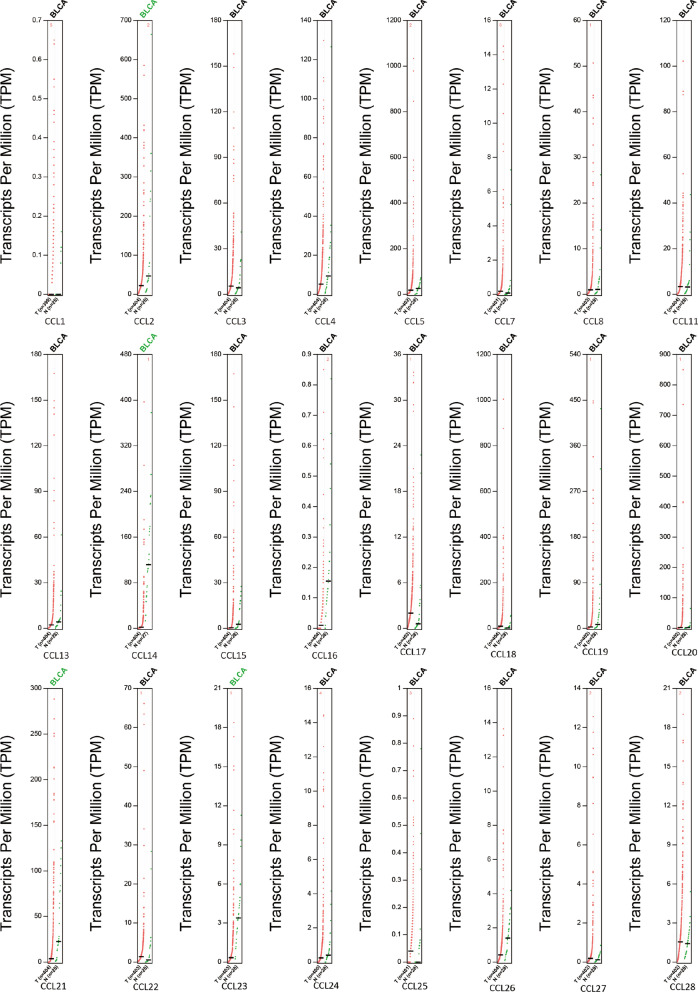
The expression of CC chemokines in BC (GEPIA)

**Fig. 3:**
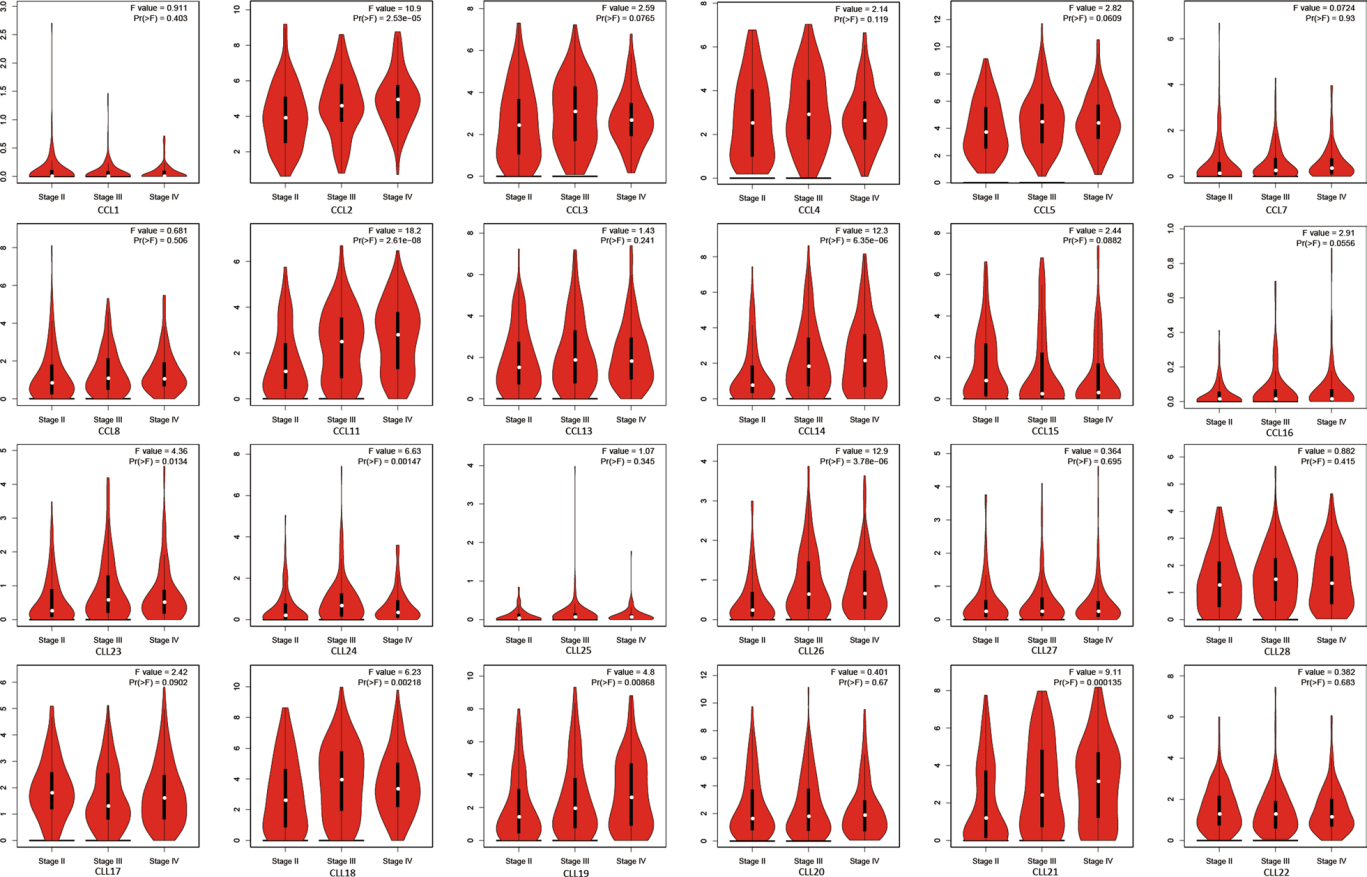
Correlation between CC chemokines and tumor stage in BC patients (GEPIA)

A total of 24 CC chemokines were retrieved using the ONCOMINE database. The distinct expression of 24 CC chemokines in BC were explored. As presented in Fig. 1, the mRNA expression levels of CCL2, CCL3, CCL4, CCL5, CCL13, CCL14, CCL19 and CCL21 were significantly reduced in BC patients. But significantly increased CCL13 (*P* = 0.011, Fold change = 2.654) were also observed in BC compared with normal tissue. Similar results were found when we assessed the transcriptional levels of CC chemokines using the GEPIA database, CCL2, CCL14, CCL21 and CCL23 were lower in BC patients than normal patients (Fig. 2). We then evaluated the correlation between the expression of differentially expressed CC chemokines and the pathological stage of BC patients. The results revealed that CCL2 (Pr = 2.53e−05), CCL11 (Pr = 2.61e−08), CCL14 (Pr = 6.35e−06), CCL18 (Pr = 0.00218), CCL19 (Pr = 0.00868), CCL21 (Pr = 0.000135), CCL23 (Pr = 0.0134), CCL24 (Pr = 0.00147) and CCL26 (Pr = 3.78e-06) may play a significant role in the tumorigenesis in BC patients (Fig. 3).

## Prognostic value of the mRNA expression of CC chemokines in BC patients

**Fig. 4:**
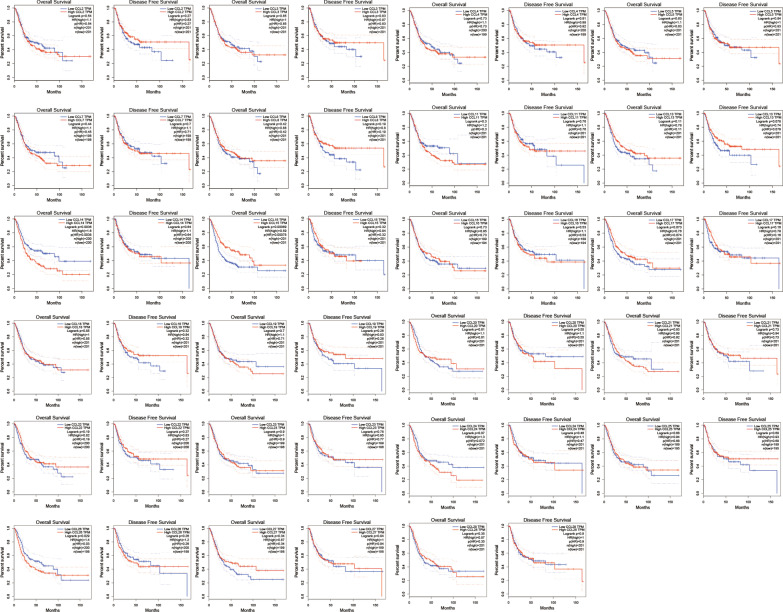
The prognostic value of mRNA level of CC chemokines in BC patients (GEPIA)

We explored the value of differentially expressed CC chemokines in the progression of BC patients. According to the data from GEPIA (not including CCL1), patients with higher levels of CCL14 (*P* = 0.0036) (Fig. 4) showed shorter overall survival (OS), but OS tended to be longer in patients with higher levels of CCL15 (*P* = 0.00069) (Fig. 4). And current results did not show a significant relation between overall survival (OS) or Disease-free survival (DFS) and other CC chemokines.

**Fig. 5:**
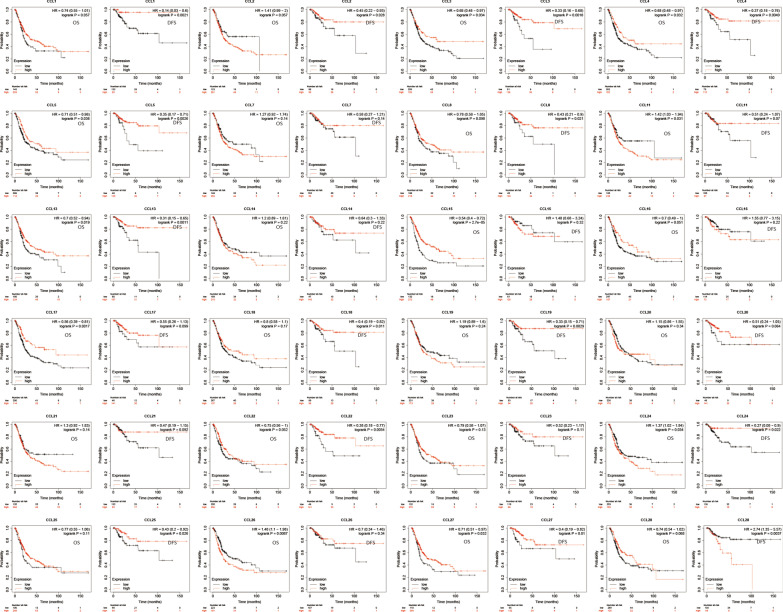
Prognostic value of CC chemokines in BC Patients (Kaplan–Meier plotter)

Besides, we also analysed the prognostic values of CC chemokines using Kaplan–Meier plotter in BC patients (Fig. 5). Significantly increased OS and DFS were observed in patients with higher levels of CCL3, CCL4, CCL5, CCL13 or CCL27. Patients with higher levels of CCL1, CCL2, CCL8, CCL18, CCL19, CCL22, CCL24, or CCL25 were associated with increased DFS and higher levels of CCL15, CCL17 were correlated with longer OS. However, there is a significant negative correlation between CCL11, CCL24, CCL26 with OS and between CCL28 with DFS.

## Genetic alteration, expression, and interaction analyses of CC chemokines in BC patients

**Fig. 6:**
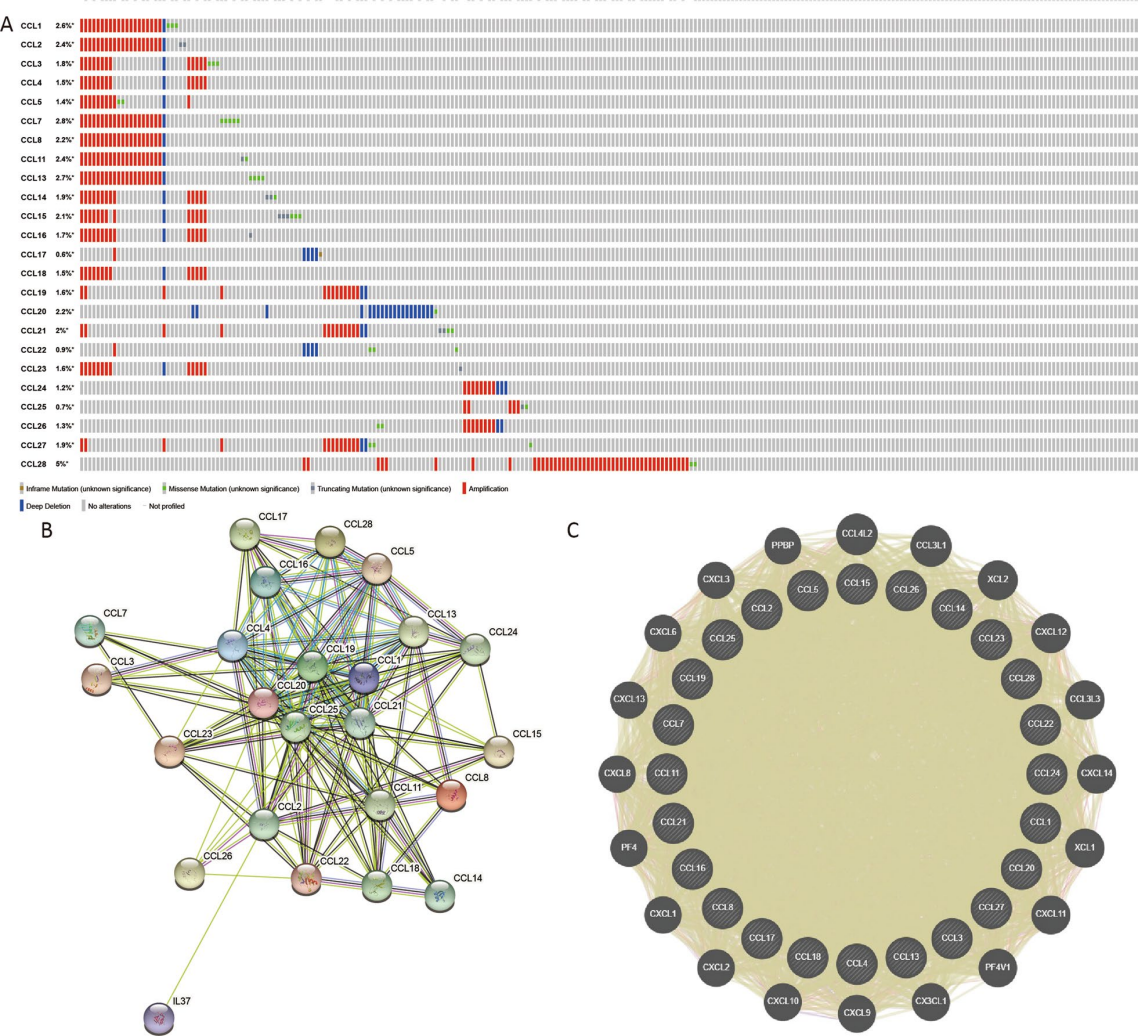
CC chemokines gene mutation and expression analyses in BC (cBioPortal, GeneMANIA and STRING). **A** Summary of alterations in different expressed CC chemokines in BC. **B**, **C** Protein–protein interaction network of different expressed CC chemokines

We assessed the genetic alterations of CC chemokines in BC patients by using the eBioPortal online tool. CCL1, CCL2, CCL3, CCL4, CCL5, CCL7, CCL8, CCL11, CCL13, CCL14, CCL15, CCL16, CCL17, CCL18, CCL19, CCL20, CCL21, CCL22, CCL23, CCL24, CCL25, CCL26, CCL27 and CCL28 were altered in 2.6, 2.4, 1.8, 1.5, 1.4, 2.8, 2.2, 2.4, 2.7, 1.9, 2.1, 1.7, 0.6, 1.5, 1.6, 2.2, 2, 0.9, 1.6, 1.2, 0.7, 1.3, 1.9 and 5% of the queried BC samples, respectively (Fig. 6A). Furthermore, we conducted a PPI network analysis of the differentially expressed CC chemokines with STRING to evaluate the potential interactions among them. As illustrated in Fig. 6B, 157 edges and 24 nodes were obtained. The CC chemokines were mainly associated with chemokine signaling pathway and immune cell regulation. The GeneMANIA results revealed that these CC chemokines were related to cytokine activity, chemokines receptor binding, chemotaxis immune cell migration (Fig. 6C).

## Immune cell infiltration of CC chemokines in BC patients

**Fig. 7:**
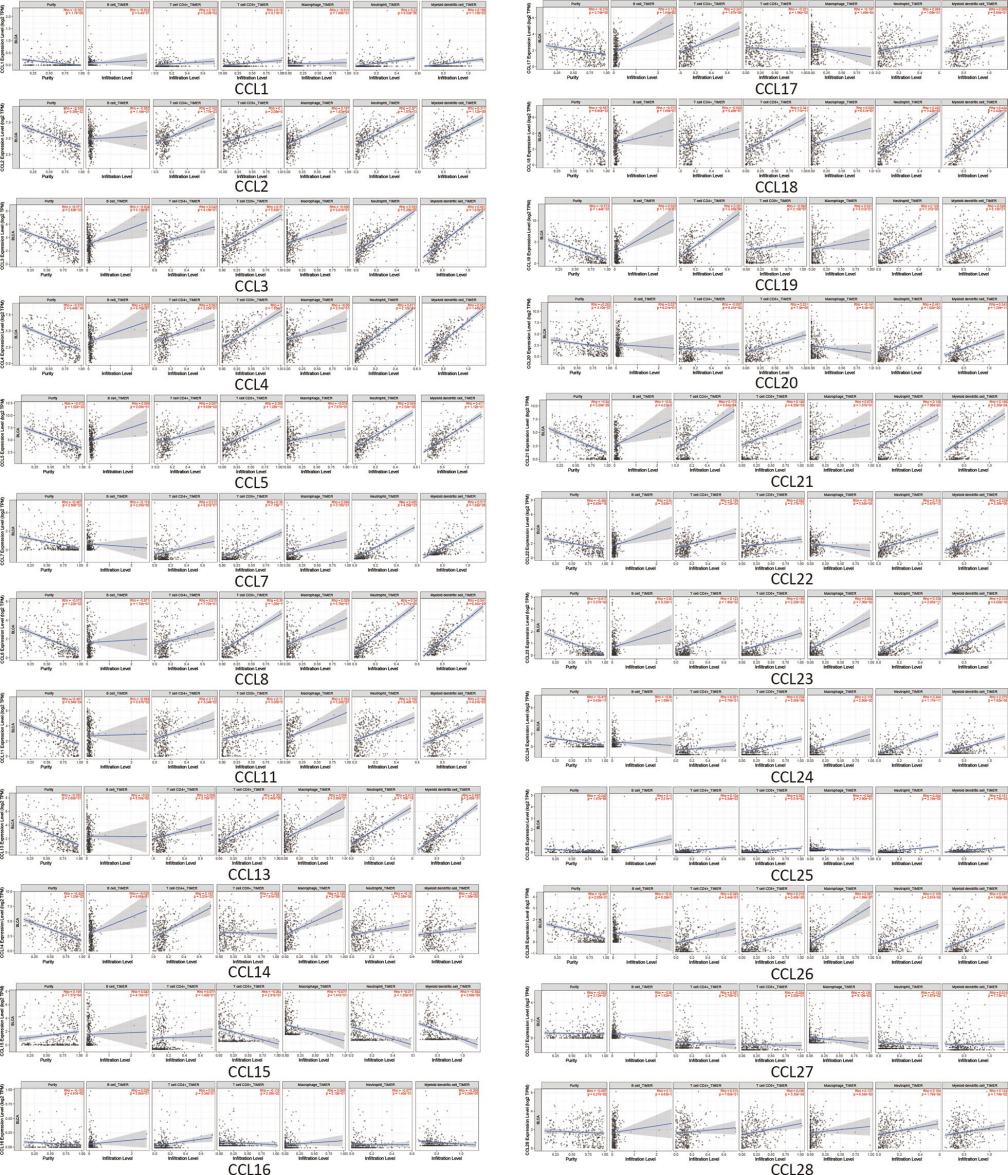
Correlations between differentially expressed CC chemokines and immune cell infiltration (TIMER). Correlations between the abundance of immune cells and the expression of CCL1-28

To explore the relation between immune cell level and cancer cell, the TIMER database are used to perform an analysis. The results (Fig. 7) indicated a high correlation between immune cell infiltration and CC chemokines as follows: CCL1 with CD8^+^ T cells (ρ = 0.10, *P* = 4.71e−02), neutrophils (ρ = 0.23, *P* = 8.53e−06), and dendritic cells (ρ = 0.168, *P* = 1.19e−03); CCL2 with CD4^+^ T cells (ρ = 0.162, *P* = 1.77e−03), CD8^+^ T cells (ρ = 0.1, *P* = 2.08e−03), macrophages (ρ = 0.197, *P* = 1.47e−04), neutrophils (ρ = 0.371, *P* = 1.87e−13), and dendritic cells (ρ = 0.311, *P* = 1.12e−09); CCL3 with neutrophils (ρ = 0.583, *P* = 6.38e−35), and dendritic cells (ρ = 0.601, *P* = 1.61e−37); CCL4 with CD8^+^ T cells (ρ = 0.4, *P* = 1.50e−15), neutrophils (ρ = 0.671, *P* = 2.12e−49), and dendritic cells (ρ = 0.601, *P* = 1.45e−37); CCL5 with CD8^+^ T cells (ρ = 0.359, *P* = 1.26e−12), neutrophils (ρ = 0.549, *P* = 2.53e−30), and dendritic cells (ρ = 0.471, *P* = 1.12e−21); CCL7 with B cell (ρ = − 0.119, *P* = 2.25e−02), CD8^+^ T cells (ρ = 0.362, *P* = 7.78e−13), neutrophils (ρ = 0.485, *P* = 4.26e−23), macrophages (ρ = 0.517, *P* = 1.62e−26); CCL8 with CD8^+^ T cells (ρ = 0.388, *P* = 1.06e−14), neutrophils (ρ = 0.54, *P* = 2.71e−29), and dendritic cells (ρ = 0.541, *P* = 2.45e−29); CC11 with CD4^+^ T cells (ρ = 0.112, *P* = 3.24e−02), CD8^+^ T cells (ρ = 0.11, *P* = 3.05e−02), macrophages (ρ = 0.263, *P* = 3.20e−07), neutrophils (ρ = 0.158, *P* = 2.40e−03), and dendritic cells (ρ = 0.146, *P* = 4.91e−03); CCL13 with CD8^+^ T cells (ρ = 0.302, *P* = 3.46e−09), neutrophils (ρ = 0.412, *P* = 1.70e−16), and dendritic cells (ρ = 0.468, *P* = 2.00e−21); CCL14 with CD4^+^ T cells (ρ = 0.153, *P* = 3.21e−03), CD8^+^ T cells (ρ = − 0.204, *P* = 7.81e−05), macrophages (ρ = 0.188, *P* = 2.78e−04), neutrophils (ρ = − 0.19, *P* = 2.38e−04), and dendritic cells (ρ = − 0.225, *P* = 1.36e−05); CCL15 with CD8^+^ T cells (ρ = − 0.264, *P* = 2.91e−07), neutrophils (ρ = − 0.271, *P* = 1.25e−07), and dendritic cells (ρ = -0.304, *P* = 2.68e-09); CCL16 with CD8^+^ T cells (ρ = − 0.118, *P* = 2.39e−02) and dendritic cells (ρ = − 0.209, *P* = 5.56e−05); CCL17 with B cell (ρ = 0.123, *P* = 1.84e−02), CD4^+^ T cells (ρ = 0.247, *P* = 1.67e−06), CD8^+^ T cells (ρ = − 0.221, *P* = 1.96e−05), and macrophages (ρ = − 0.197, *P* = 1.46e−04); CCL18 with CD8^+^ T cells (ρ = 0.341, *P* = 1.71e−11), neutrophils (ρ = 0.432, *P* = 3.42e−18), and dendritic cells (ρ = 0.434, *P* = 2.42e−18); CCL19 with CD4^+^ T cells (ρ = 0.281, P = 4.35e−08) and neutrophils (ρ = 0.126, *P* = 1.37e−02); CCL20 with CD8^+^ T cells (ρ = 0.231, *P* = 7.9e−06), macrophages (ρ = − 0.141, *P* = 6.8e−03), neutrophils (ρ = 0.461, *P* = 1.02e−20), and dendritic cells (ρ = 0.343, *P* = 1.25e−11); CCL21 with CD4^+^ T cells (ρ = 0.173, *P* = 8.64e−04), CD8^+^ T cells (ρ = 0.149, *P* = 4.20e−03), neutrophils (ρ = 0.138, *P* = 7.96e−03), and dendritic cells (ρ = 0.186, *P* = 3.35e−04); CCL22 with CD4^+^ T cells (ρ = 0.189, *P* = 2.72e−04), macrophages (ρ = −0.179, *P* = 5.54e−04), neutrophils (ρ = 0.316, *P* = 5.67e−10), and dendritic cells (ρ = 0.239, *P* = 3.39e−06); CCL23 with CD4^+^ T cells (ρ = 0.123, *P* = 1.86e−02), CD8^+^ T cells (ρ = 0.159, *P* = 2.22e−03), neutrophils (ρ = 0.338, *P* = 2.85e−11), and dendritic cells (ρ = 0.381, *P* = 4.56e−10); CCL24 with CD8^+^ T cells (ρ = 0.234, *P* = 5.56e−06), macrophages (ρ = 0.116, *P* = 2.60e−02), neutrophils (ρ = 0.344, *P* = 1.17e−11), and dendritic cells (ρ = 0.275, *P* = 7.82e−08); CCL25 with B cell (ρ = 0.152, *P* = 3.51e−03), CD4^+^ T cells (ρ = 0.104, *P* = 4.54e−02), neutrophils (ρ = 0.244, *P* = 2.19e−06), and dendritic cells (ρ = 0.151, *P* = 3.79e−03); CCL26 with B cell (ρ = − 0.207, *P* = 6.30e−05), CD8^+^ T cells (ρ = 0.218, *P* = 2.49e−05), macrophages (ρ = 0.267, *P* = 1.99e−07), neutrophils (ρ = 0.189, *P* = 2.67e−04), and dendritic cells (ρ = 0.247, *P* = 1.66e−06); CCL27 with macrophages (ρ = − 0.136, *P* = 9.10e−03) and neutrophils (ρ = − 0.133, *P* = 1.07e−02); CCL28 with B cell (ρ = 0.103, *P* = 4.83e−02), CD8^+^ T cells (ρ = 0.186, *P* = 3.35e−04), macrophages (ρ = 0.137, *P* = 8.34e−03), neutrophils (ρ = 0.194, *P* = 1.76e−04), and dendritic cells (ρ = 0.124, *P* = 1.74e-02).

## Discussion

Bladder cancer is one of the most common causes of cancer-related deaths worldwide [24]. CC chemokines, which can be expressed by tumor cells and other cells, play an important role in the immune cell tumor trafficking [25–27], tumor metastasis [28] and apoptosis [29]. Accumulating evidence has revealed the potential value of CC chemokines in cancer immunotherapy. However, the prognostic and possible therapeutic value of CC chemokines in BC is not yet defined.

Among the CC chemokines, CCL2 is the most studied in BC. Expression of CCL2 was higher in BC tissues and in human BC cell lines [30]. And this trend was obvious with the stage of BC [30]. The reduced expression of CCL2 downregulated by miR-1-3p could inhibit the metastasis and proliferation of BC cells [30]. Besides, animal experiment also proved the increased CCL2 expression in murine bladder cancer cell line [31]. Recent studies have revealed a negative relationship between prognosis, survival and CCL2 in BC patients received chemotherapy [16, 32]. While gemcitabine-treated BC cells also induced more CCL2 which may recruit more monocyte-myeloid-derived suppressed cells (M-MDSCs) and incurred poor prognosis [33]. Several studies demonstrated overexpression of CCL2 in bladder cancer was correlated with tumor invasion, tumor progression [34] and lymphatic metastasis [35]. HSP47 [36], LNMAT1 [35], ERβ [37] seem to be related to CCL2 directly or indirectly. While in this study, the results indicated that the expression level of CCL2 was reduced in BC than normal sample. Moreover, a low CCL2 expression was significantly correlated with poor DFS.

For the other CC chemokines, a previous study demonstrated that CCL1 can be up-regulated by estrogen receptors alpha and then enhance bladder cancer cell invasion [38]. CCL1/CCR axis was found to be correlated with cancer-related inflammation and immune evasion [39]. Besides, GAS5 may inhibit bladder cancer cell proliferation by suppressing the expression of CCL1 [40]. However, our results did not reveal a significant difference in CCL1 expression between BC and normal patients. In vitro experiments found that upregulated CCL3 inhibits the immune response which would favor tumor growth [31]. Interestingly, a higher CCL3 expression seems to be correlated with a better OS and DFS in our study. Steve et al. [41] found CCL18 was significantly increased in voided urine of BC, but it seems not related with bladder cancer grade nor stage [42]. There are studies showed CCL18 may enhance migration and invasion by binding CCR8 in bladder cancer cells [43]. According to our study, there is no difference in CCL18 expression between normal patients and BC. Different from previous studies, patients with a higher level of CCL18 are associated with a better DFS in this study. Feng et al. [44] found the CCL21/CCR7 axis promotes migration and invasion capacity of urinary bladder cancer cells and induces lymphatic metastatic spread. In the present study, CCL21 did not show a significant influence on OS or DFS. We also found the expression level of CCL4/5/14/19/21/23 were lower in BC patients than normal patients. Whereas in certain data sets CCL13 was increased significantly in BC patients compared with normal patients. Various means of data collection in different studies may be the reason for differentially expressed CCL13. In addition, CCL2, CCL11, CCL14, CCL18, CCL19, CCL21, CCL23, CCL24, CCL26 were markedly related with clinical stage in BC patients and CC chemokines were related to cytokine activity, chemokines receptor binding, chemotaxis immune cell migration. In this study, we found a significant correlation between the expression of CC chemokines and the infiltration of the immune cell types, indicating that CC chemokines may also play a significant role in the immune activity.

One of the limitations of our study was that a detailed description or stratified analysis was missed about characteristics of the patients and clinical subtypes of BC. The data in our study are extracted from several online databases and different studies. Particularly, part of the clinical course of bladder tumors, characteristics of the patients baseline data is not complete in these studies. Among these patients, stage Ois bladder urothelial carcinoma, superficial bladder cancer, infiltrating bladder urothelial carcinoma are included. Therefore, a detailed description or stratified analysis was missed about these items related to diagnosis or therapeutic. Moreover, in the Figs. 4 and 5, several of the survival curves cross each other, which limit the usefulness of the log-rank test for comparing the survival outcome in our study. Further analysis, like Parametric Regressive Model, should be perform to compare it. Regrettably, we failed to establish a univariate- and multivariable Cox proportional-hazards model due to the fact that some valid information may be missed. What`s more, it`s a limitation of our study that adjusted p-value was not employed to prevent family-wise error rates.

## Conclusions

In this research, we analyzed the prognostic and therapeutic value of CC chemokines in BC. Our results provided the information that CC chemokines might play an important role in BC oncogenesis which indicates a potential target of BC. We hope our results provide novel insights on the therapeutic targets of BC and help clinicians make a better personal treatment plan. However, further studies are needed to elucidate the difference between subgroup of BC classified by Infiltration degree or grade of these genes in BC.

## Data Availability

The datasets generated and/or analysed during the current study are available in the dataset as presented in the methods section.

## Abbreviations

BC: Urothelial bladder cancer
GEPIA: Gene Expression Profiling Interactive Analysis
TURBT: Transurethral resection of bladder tumour
RFS: Recurrence-free survival
OS: Overall survival
TCGA: The Cancer Genome Atlas
PPI: Protein–protein interaction
DFS: Disease-free survival
M-MDSCs: Monocyte-myeloid-derived suppressed cells
HSP47: Heat shock protein 47
LNMAT1: Lymph Node Metastasis Associated Transcript 1
ERβ: Estrogen receptor beta
GAS5: Growth arrest‑specific 5
